# Supplementary material for: Factors responsible for Ixodes ricinus presence and abundance across a natural-urban gradient
Source: PLoS One. 2023 May 17;18(5):e0285841. doi: 10.1371/journal.pone.0285841 (PMC10191369; doi:10.1371/journal.pone.0285841)
Supplement: S1 Table — (DOCX) [file pone.0285841.s001.docx]

**S1 Table. Swedish land cover nomenclature including grid code, land cover type and definition^1^.**

| **Grid code** | **Land cover** | **Definition** |
| --- | --- | --- |
| 111 | Pine forest not on wetland | Tree-covered areas outside of wetlands with a total crown cover of >10% where >70% of the crown cover consists of pine. Trees are higher than 5 meters |
| 112 | Spruce forest not on wetland | Tree-covered areas outside of wetlands with a total crown cover of >10% where >70% of the crown cover consists of spruce. Trees are higher than 5 meters |
| 113 | Mixed coniferous not on wetland | Tree-covered areas outside of wetlands with a total crown cover of >10% where >70% of consists of pine or spruce, but none of these species are >70%. Trees are higher than 5 meters. |
| 114 | Mixed forest not on wetland | Tree-covered areas outside of wetlands with a total crown cover of >10% where neither coniferous nor broadleaved crown cover reaches >70%. Trees are higher than 5 meters. |
| 115 | Broadleaved forest not on wetland | Tree-covered areas outside of wetlands with a total crown cover of >10% where >70% of the crown cover consists of broadleaved trees (primarily birch, alder and/or aspen). Trees are higher than 5 meters. |
| 116 | Broadleaved hardwood forest not on wetland | Tree-covered areas outside of wetlands with a total crown cover of >10 where >70% of the crown cover consists of broadleaved trees, of which >50% is broadleaved forest (mainly oak, beech, ash, elm, linden, maple, cherry and hornbeam). Trees are higher than 5 meters. |
| 117 | Broadleaved forest with broadleaved hardwood forest not on wetland | Tree-covered areas outside of wetlands with a total crown cover of >10 where >70% of the crown cover consists of broadleaved trees, of which 20 - 50% is broadleaved forest (mainly oak, beech, ash, elm, linden, maple, cherry and hornbeam). Trees are higher than 5 meters. |
| 118 | Temporarily non-forest not on wetland | Open and re-growing clear-felled, storm-felled or burnt areas outside of wetlands. Trees are less than 5 meters. |
| 121 | Pine forest on wetland | Tree-covered areas on wetlands with a total crown cover of >10% where >70% of the crown cover consists of pine. Trees are higher than 5 meters |
| 122 | Spruce forest on wetland | Tree-covered areas on wetlands with a total crown cover of >10% where >70% of the crown cover consists of spruce. Trees are higher than 5 meters |
| 123 | Mixed coniferous on wetland | Tree-covered areas on wetlands with a total crown cover of >10% where >70% of consists of pine or spruce, but none of these species are >70%. Trees are higher than 5 meters |
| 124 | Mixed forest on wetland | Tree-covered areas on wetlands with a total crown cover of >10% where neither coniferous nor broadleaved crown cover reaches >70%. Trees are higher than 5 meters |
| 125 | Broadleaved forest on wetland | Tree-covered areas on wetlands with a total crown cover of >10% where >70% of the crown cover consists of broadleaved trees (primarily birch, alder and/or aspen). Trees are higher than 5 meters |
| 126 | Broadleaved hardwood forest on wetland | Tree-covered areas on wetlands with a total crown cover of >10 where >70% of the crown cover consists of broadleaved trees, of which >50% is broadleaved forest (mainly oak, beech, ash, elm, linden, maple, cherry and hornbeam). Trees are higher than 5 meters. |
| 127 | Broadleaved forest with broadleaved hardwood forest on wetland | Tree-covered areas on wetlands with a total crown cover of >10 where >70% of the crown cover consists of broadleaved trees, of which 20 - 50% is broadleaved forest (mainly oak, beech, ash, elm, linden, maple, cherry and hornbeam). Trees are higher than 5 meters. |
| 128 | Temporarily non-forest on wetland | Open and re-growing clear-felled, storm-felled or burnt areas on wetlands. Trees are less than 5 meters |
| 2 | Open wetland | Open wetland Open land where the water for a large part of the year is close by, in or just above the ground surface |
| 3 | Arable land | Agricultural land used for plant cultivation or kept in such a condition that it can be used for plant cultivation. The land should be able to be used without any special preparatory action other than the use of conventional farming methods and agricultural machinery. The soil can be used for plant cultivation every year. Exceptions can be made for an individual year if special circumstances exist. |
| 41 | Non-vegetated other open land | Other open land that is not wetland, arable land or exploited vegetation-free surfaces and has less than 10% vegetation coverage during the current vegetation period. The ground can be covered by moss and lichen. |
| 42 | Vegetated other open land | r open land Other open land that is not wetland, arable land or exploited vegetation-free surfaces and has more than 10% vegetation coverage during the current vegetation period. |
| 51 | Artificial surfaces, building | A durable construction consisting of roofs or roofs and walls and which is permanently placed on the ground or partly or wholly below ground or is permanently placed in a certain place in water and is intended to be designed so that people can stay in it. |
| 52 | Artificial surfaces, not building or road/railway | Artificial open and vegetation-free surfaces that are not building or road/railway. |
| 53 | Artificial surfaces, road/railway | Road or railway. |
| 61 | Inland water | Lakes or watercourses. |
| 62 | Marine water | Sea, ocean, estuaries or coastal lagoons. |

^1^ Swedish Environmental Protection Agency. Svenska Marktäckedata. Edition 2. 2 [Internet]. 2019. Available from: [https://www. naturvardsverket. se/upload/sa-mar-miljon/kartor/NMD-Produktbeskr-NMD2018Basskikt-v2-2. pdf](https://www.naturvardsverket.se/upload/sa-mar-miljon/kartor/NMD-Produktbeskr-NMD2018Basskikt-v2-2.pdf)
