# Supplementary material for: Factors responsible for Ixodes ricinus presence and abundance across a natural-urban gradient
Source: PLoS One. 2023 May 17;18(5):e0285841. doi: 10.1371/journal.pone.0285841 (PMC10191369; doi:10.1371/journal.pone.0285841)
Supplement: Attachment — Submitted filename: Response to reviewers.docx [file pone.0285841.s002.docx]

PLOS ONE

October 21, 20222

Re: Factors responsible for *Ixodes ricinus* abundance across an urbanization gradient

PONE-D-22-15726

**Dear reviewers,**

We have read your comments carefully and tried our best to address them one by one. Please see answers your comments below. All modification in the manuscript have been highlighted in yellow.

Kindest regards,

Corresponding author

Thérese Janzén

PhD student

[therese.janzen@sh.se](mailto:therese.janzen@sh.se)

Phone +46 8 608

Södertörn University, School of Natural Sciences, Technology and Environmental Studies

SE-141 89 Huddinge, Sweden

Responses to reviewer #1 comments:

Line 101: Note that "mopping" is modified dragging technique, as dragging is much better known.

*Response: We have added information reading our mopping technique to the manuscript (lines 146-149).*

Line 109: I did not see any details about the frequency of collections from each site per season. How frequently were sites visited per year? The large number of larvae and relatively small number of adults (lines 198 - 199) could indicate undersampling during peak nymph/adult activity.

*Response: We have added details regarding the frequency of our collections to the manuscript and explanations to the capture ratios for the different life stages (lines 154-158).*

Lines 250 - 285: A lot of information is provided here that recites what can be read in the tables. Written text here would be best used for highlighting the patterns to help draw the reader's eye to particular areas of the table. There are several ways I can suggest to consider organizing the text: 1) organize by broad general patterns related to land cover (e.g. lines 244 - 245, mixed forest) vs. unusual patterns (e.g. lines 257 - 258, broadleaved with hardwood, and 268 - 269, wetlands) or 2) organize by positive effects vs. negative effects or 3) organize by strength of the association, i.e. coefficients. The text explanation of these results should help aid in understanding or highlighting parts of the tables presented.

*Response: Thank you very much for pointing this out. We have revised this section of the result section. We took you advice and organized by broad general patterns with focus on the effects of different land cover types on occurrence and abundance of* Ixodes Ricinus *(lines 291-333).*

Line 324 -326: Vegetation height for adults in particular could be biased by a small collection (n = 13). Much more variation might be seen if more adults were collected.

*Response: Our systematic random sampling model with data collected from many different sites produced data on presence and absence of ticks which is crucial information when analyzing factors responsible for* I. ricinus *distribution. This sampling method yields mean number of ticks per four square meter which allows for comparisons between sites, within this study as well as with other studies using similar procedures. In addition, in the statistical analyses adults and nymphs were analyzed together (n=317+13=330).*

Line 377: Is this a reference to the wetlands results? Please clarify

*Response: Thank you for your question. We have edited this sentence and clarified that we are discussing the land cover type Open water (lines 431-433).*

Line 322: "tick's" should be changed to "ticks"

*Response: Revised accordingly (line 372).*

Line 330: grass

*Response: Revised accordingly (line 380).*

Line 332: change "is indicating" to indicates

*Response: Thank you for pointing this out. We have revised this sentence (line 384-385).*

Line 342: represents

*Response: Revised accordingly (line 392).*

Line 372: Revise this sentence, "absolutely most ticks" is awkward

*Response: Thank you very much for pointing this out. We have revised this sentence (line 406-407).*

Line 388: measures

*Response: We have revised this sentence (lines 441-443).*

Line 406 - 407: Incomplete sentence

*Response: Thank you very much for pointing this out. We have revised this sentence (lines 460-462).*

Line 412: compared

*Response: Revised accordingly (line 470).*

Final note: Given the author's stated interest in tick-borne disease surveillance, life stages relevant to transmission should be included in larger numbers. Typically, researchers look at density of infected nymphs (DIN) to discuss human risk of exposure to disease. As the authors noted, the clustering of larvae interfered with the analysis, but larvae are also over represented in this study especially given that this is a life stage that generally poses less disease risk to humans and animals.

Limited collection of adults (n = 13) compared to larvae (n = 992) is a concern because some major tick-borne disease, e.g. Borrelia spp. infections, are typically transmitted by adults and nymphs only. Maternal transmission of Borreliae to eggs/larvae does not occur, so unfed larvae are not considered a disease risk for Borrelia. Thus, targeting adult and nymph life stages in collections is imperative to understand the whole system.

Although the authors here are not considering infection in ticks, I recommend looking more closely at literature in which DIN is calculated and used to interpret exposure risk.

In one paper, Jouda et al. 2004, the authors collected and analyzed 108 - 145 nymphs per altitude class, per year, with a total of 1112 nymphs included in the study. Likewise, 9 - 149 adults were included per altitude class, per year, for a total of 507 adults across three years. The authors should seek to collect comparable numbers, particularly when evaluating such a large collection of environmental variables for their effect on densities.

I applaud the authors for the spatial study design and analyses. The limited and skewed tick collections, unfortunately undermine the results overall, and the applicability of this data to understand human exposure risk.

*Response: We were pleased about the kind words about our spatial study design and analyzes. We believe that this sampling method is important both for comparison between sites as well as with other studies using similar procedures.*

*In our spatial analysis design, tick absence is as important as tick presence both for assessing the prevalence of ticks along the urbanization gradient and to understand how different habitats effects the occurrence of ticks in different areas.*

Responses to reviewer #2 comments:

The overall value of presented study is high and the outcome may have additional value in tick-borne diseases ecoepidemiology and use as starting point in landscape planning and urban planning related studies, risk measurements for public health. The strength of the manuscript and presented study is solid basis of up-to date literature, with substantial representation of papers describing local environment characteristics that allowed for good study design. Manuscript is not free from minor redaction issues and few major gaps, yet this should be easily improved by the Authors.

*Response: We also believe that the overall value of our study is high and with the comments and suggestions from both reviewers we hope that the manuscript will be accepted for publication.*

Title: please specify which ticks! Also, In Reviewer's opinion it's about risk factors, not risks in it's current state.

*Response: Thank you very much for pointing this out. We have changed the aim of the paper from risks to possible factors responsible for* Ixodes ricinus *abundance across an urbanization gradient in Northern Europe (lines 67-68).*

9-10 – broad thesis, please provide examples of epidemiological studies

*Response: We have added the following reference to this statement (line 30):*

*Vanwambeke SO, Linard C, Gilbert M. Emerging challenges of infectious diseases as a feature of land systems. Current Opinion in Environmental Sustainability. 2019 Jun 1;38:31-6.*

*In this article, the authors are discussing how various environmental processes including land cover and land use affects infectious diseases and especially vector-borne diseases. Land cover and land use connects vectors, hosts and humans, including different activities exposing them to pathogens.*

*Human-environment interactions are therefore crucial components of the epidemiological patterns of vector-borne diseases.*

9-17 – Phrases like: „. Greenspaces of all kinds are crucial for terrestrial biodiversity” or „unwanted insects” or „are not only annoying” sounds maybe catchy for lay-man, but are biased, empty and not suitable for scientific journal.

Thus I strongly advise to review and adjust the language used.

*Response: Thank you very much for pointing this out. We have revised this section (lines 34-37).*

15 „arachnoids” (arachnids).

*Response: Revised accordingly (line 35).*

17 – what inf. Dis.? This statement lacks of examples of vector borne diseases of epidemiological importance for precision.

*Response: We don’t think so since vector-borne diseases are not the focus of this paper. To be clearer we have revised this sentence and added references (lines 35-37).*

19 – how long, what is long in tick lifecycle?

*Response: Thank you for your question. We revised this sentence (lines 39-40).*

24 – rephrase (ticks surely are not hoping for anything)

*Response: Thank you for this nice reminder. We have revised this sentence (lines 49-50).*

34-38 – in reviewer’s opinion it is important to underline that these descriptions are for I. ricinus, because other ticks of medical importance, Dermacentor reticulats, have different (almost opposite) patterns of occurence, described i.a. in vast environmental studies performed by Mierzejewska et al.

*Response: Thank you for pointing this out. We have revised the manuscript and underlined that these descriptions are for* I. ricinus *ticks (lines 56-60).*

40 – like above, specify which ticks. It is worth to specify in discussion as well.

*Response: Revised accordingly.*

43-46 – please ellaborate importance of nymphs, within introduction, before the aims of the project. As for now such insert is confusing.

*Response: Thank you for pointing this out. We have added information regarding the distribution of* I. ricinus *life stages and importance of nymphs to the introduction (lines 41-47).*

56 – latin radius, please try rephrase using a singular nom. for clarity throughout the ms.

*Response: Revised accordingly*

Fig 1. Please state if inventory plot 2x2 is an example photography or satellite view. It is unclear (not easily distinguishable what is presented in the circle picture). Maybe arrows with description would be helpful.

*Response: Thank you very much for pointing this out. We have stated that is a photography from one of our collection sites and added descriptions to what is presented in the picture.*

80 – please provide reference to urbanization index

*Response: A common way of calculating an urbanization index is to use the proportion of artificial areas in a surrounding area. We have in addition, extracted open water since Stockholm County is surrounded by water (lines 111-120).*

98 – is this the description of the snowball technique? Could you please confirm if that means that the compass arrow pointed both distance between 0-36 m as 1degree to 10 cm, as well as the direction? This randomization technique seems interesting and I feel it was not covered enough.

*Response: Thank you very much for your nice comment. We have added more information about our systematic random sampling technique to the method section (lines 137-145).*

101-106 – please state if were collected ticks cathegorized at site or later?

*Response: Thank you for your nice reminder. We have added to the manuscript that the ticks were categorized in the laboratory as well (line 151-152).*

107-112. In line 88 you stated that there were 35 Sites monitored by checking five 2x2 inventory plots. In lines 107-112 you stated that sampling were performed in 8h period of the day for ALL 35 sites at the same day, is that correct? Or do you mean that in each Site sampling was performed within same day? How many timeas each Site was sampled? Please clarify sampling as this is not clear for now, consider adding some suppl. materiały e.g tables.

*Response: Thank you very much for your questions. We sampled about three sites per day from different gradients of urbanization, either it the direction from more rural to urban or from more urban to rural. We have added information regarding our sampling method to this section of the manuscript (lines 127-131, 154-158).*

299 – this is probably Fig 3 description?

*Response: Revised accordingly (line 348).*

Aim of the study was to investigate the risk of tick encounters (not done?)

The tool was to identify the potential risk factor (success).

*Response: Thank you very much for pointing this out. We have changed our aim to possible factors responsible for* I. ricinus *presence and abundance across an urbanization gradient.*

Table 4? I think this is actually risk factors, not risks. How were those risks valued? Is it solely on the strength of effects? Or is there any formula used?

*Response: Thank you very much for pointing this out. We have changed from ‘risk factors’ to ‘factors responsible for* I. ricinus *abundance across an urbanization gradient’.*

303: risk factors rather than risk, suggestion: it is worth considering explaining in methods that you intended to evaluate risk on the basis of tick presence/abundance in reference to any detected risk factors. For now ms is lacking risk measurement description, it is only resulting from discussion part and it's virtually about risk factors.

*Response: We have explained in the methods section that we analyzed possible risk factors for I. ricinus presence and abundance (lines 67-68, 77-78, Table 4).*

406-407 - for rephrasing, seem like different kind of journalism

*Response: Thank you for this kind reminder. We have revised this sentence (lines 460-462).*

It seems logical that risk is affected by positive or negative effects, however risk itself was not described in my opinion. E.g., what is the matching value for statement that "there is still a considerable risk for tick encounters in highly urbanized areas.". What risk level is considerable and what is not?

*Response: Thank you very much for pointing this out. We have changed from ‘risk factors’ to ‘factors responsible for* I. ricinus *abundance across an urbanization gradient’.*

We hope that our manuscript is now ready for publication in PLOS ONE.
