# Supplementary material for: Factors responsible for Ixodes ricinus presence and abundance across a natural-urban gradient
Source: PLoS One. 2023 May 17;18(5):e0285841. doi: 10.1371/journal.pone.0285841 (PMC10191369; doi:10.1371/journal.pone.0285841)
Supplement: Attachment — Submitted filename: Response to reviewer.docx [file pone.0285841.s003.docx]

PLOS ONE

January 21, 2023

Re: Factors responsible for *Ixodes ricinus* presence and abundance across a natural-urban gradient

PONE-D-22-15726R1

**Dear Janice L. Bossart,**

thank you for the opportunity to revise our manuscript. We appreciate you and the reviewers for your time in reviewing our paper and providing valuable comments. We have considered the comments and tried our best in addressing every one of them. Please see answers to all comments below. All modification in the manuscript have been highlighted in yellow.

Kindest regards,

Corresponding author

Thérese Janzén

PhD student

[therese.janzen@sh.se](mailto:therese.janzen@sh.se)

Phone +46 8 608 4000

Södertörn University, School of Natural Sciences, Technology and Environmental Studies

SE-141 89 Huddinge, Sweden

**Response to main comments**

We agree with the reviewers that our manuscript on ticks in urban areas and the risk they pose to humans are pets are important to investigate. The comments and suggestions from the editor and the reviewers have been a great help in improving our manuscript. We have started with answering the main comments raised from the editor.

Our sampling methods were designed to estimate the presence and abundance of *Ixodes ricinus* along the natural-urban gradient in Stockholm County, Sweden. First, regarding our experimental design we have made changes and clarifications throughout the manuscript. Second, regarding the difference in sampling effort (5 or 10 plots) we have here as well, clarified our methods and explained that we are analyzing tick presence and abundance in the individual plots how the factor *Site* have been used in the statistical models to adjust for the difference in sampling effort at the different sites.

Third, regarding the number of adults collected in this study it is our understanding that in the natural tick populations, there are much fewer adults compared to larvae and nymphs (Dobson et al., 2011; Ruiz-Fons et al., 2012). This is due to the longevity of *I. ricinus* ticks and that a large amount of time is spent of off the host, where the environment is posing a threat of survival of individual ticks (Mejlon, 2000). We have removed the adults from all the statistical models and the focus is now on the presence and abundance of larvae and nymphs. In addition, we have clarified in the manuscript how sampling was conducted across sites and time.

Our revised manuscript is now clearer!

We have also included a point-by-point response to the reviewer´s specific comments below.

**Dear reviewer,**

We have read your comments carefully and tried to address them one by one. Please see answers to your comments below. All modification in the manuscript have been highlighted in yellow.

Kindest regards,

Corresponding author

Thérese Janzén

PhD student

[therese.janzen@sh.se](mailto:therese.janzen@sh.se)

Phone +46 8 608 4000

Södertörn University, School of Natural Sciences, Technology and Environmental Studies

SE-141 89 Huddinge, Sweden

**Response to Reviewer #3 comments**

This is a useful investigation into what local site and landscape level factors influence the abundance of Ixodes scapularis in an urban landscape. Importantly, this study highlights the fact that ticks can occur in urbanized areas, and thus may pose a risk to human and companion animal health. Nevertheless, there are several major and minor shortcomings of this study.

*Response: We also believe that this is an important investigation into both local site and landscape level factors influencing tick presence and abundance in urban landscape. We have addressed all of the comments which helped us improving our manuscript*.

The use of the term “rural-urban gradient”. This term does not accurately reflect the landscape variables tested in this study. Non-urban (aka. artificial surface) doesn’t necessarily mean rural.

*Response: Thank you for pointing this out. We have revised the terminology and changed from rural-urban to natural-urban gradient.*

This study is better suited to just look at nymphs and larvae. The limited collection of adult ticks makes it difficult to make any conclusions about the adult life stage.

*Response: The adult ticks have been excluded from the statistical analyses and the focus is now on the effect of land cover composition and configuration on larval and nymphal tick presence and abundance.*

The outcome of objective 2 (effect of land cover at each sampling site) is not clear. The methods state all sampling sites were in greenspaces, but do not provide landcover information.

*Response: Since we have sampled ticks in random greenspaces along the urbanization gradient, sampling sites are variable and can therefore consist of e.g., forests, meadows, open areas, or urban parks. For local land cover information at each site, we have analyzed the proportion of the different land cover types present in the smallest buffer zone (100m).*

It seems that there was no repeat sampling of sites over time. This is a major (though not fatal) flaw in study design. To convince readers of the relevance of this data set, greater effort is needed to explain how time was factored into the modeling framework. Furthermore, the distance sampled per site (40m2 if I did my calculations correctly) falls well below sampling efforts in most studies (~1000m2 per site).

*Response: In this study we have made a trade off between the number of sampling sites and the number of sampling plots per site. To be able to have a larger n-value for sites we have a used 5 or for some sites 10 replicates of sampling plots within each site. This will increase the statistical power for explaining among site variation but the representativity of each site is of course lower than if we have had more plots per site. Our argument for using 2m × 2m plots is that we want to mop the plot with our blanket and then quickly remove ticks from the blanket before they drop off by themselves. For this the 2m × 2m plot is an ideal size. This plot size is also crucial for the possibility to measure the very local conditions like vegetation height, tree steam density. Even if tree steam density is covering a slightly larger are it always start from the sampling plot which will be impossible if the sampling area is too large. This will again be a trade off between optimal tick sampling, vegetation height and tree steam density measurements. The chosen plot size is optimal for bringing all of this together.*

*There is also a trade-off between resampling individual sites and the number of sites inventoried. We have chosen to use many sites but only visit them once. Instead, we use a second order polynomial factor of sampling time as a covariate to account for the sampling time effect. We have sampled sites at different positions along the natural-urban gradient throughout the whole sampling time frame. In this way we control the effects from our other fixed factors for time. We also regarded the sampling time to be important and have therefore kept the time factors in all final models.*

There is no explanation in the methods and results on how the response variable (abundance) was adjusted to account for the variation in sampling effort across sites. For example, density of ticks should be calculated per site and then this metric would be comparable across sites with different plot numbers.

*Response: In the statistical models, tick presence and abundance are estimated in each sampling plot. By using sampling sites as random factor in the mixed models we adjust for the correlative structure of plots within site and control the repetitive measurements at each site. This will allow us to compare sites even if they have different numbers of sampling plots. All mean values analyzed are calculated at the 2m × 2m plot size.*

Reanalysis and interpretation of the results is warranted prior to publication.

*Response: We have reanalyzed all the statistical models with only larvae and nymphs. In addition, we have reinterpreted the results and made changes accordingly in the discussion.*

Unable to find data available on the stated website. Please provide direct link.

*Response: The data will be provided when the manuscript has been accepted for publication (as recommended on the PLOS ONE website).*

Line 21: describe what “40% urbanization” is in the context of this study.

*Response new line 21: We have revised the sentence.*

*Tick abundance was higher in rural areas with large natural and seminatural habitats, but ticks were also present in parks and gardens in highly urbanized areas.*

Line 72: effect of local factors on what? effect of land cover on what? Results and discussion talk about presence/absence of ticks, but this not stated in any objectives.

*Response new line 71: Thank you for pointing this out. We have reformulated the objectives of this study.*

Line 79: remove “large” – this is a vague qualifier

*Response new line 81: Accepted*

Line 84: the area of a circle with a 100m radii would be 0.0314km2

*Response new line 88: Accepted*

Line 107: At what time of year did sampling occur in 2017? How many times were sites visited/resampled? What were the intervals of collection? How does this relate to known activity time of life stages in the region?

*Response new line 113: Sampling for both years took place from June to October. At each site we sampled 5 or 10 plots and each site were visited once. During each collection day we sampled on average three sites sampling either from more natural to urban sites or in the direction from urban to natural sites, changing the direction randomly.*

*Ticks may have both unimodal and bimodal or even multimodal activity patters for the different tick stages (Mejlon, 2000). Therefore, we have sampled ticks from June to October for both 2017 and 2019.*

Line 127: At what time of year did sampling occur in 2019? How many times were sites visited/resampled? What were the intervals of collection? How does this relate to known activity time of life stages in the region?

*Response new line 133: Please see previous comment*

Line 128: Were any of the sites sampled in 2017 resampled in 2019? It is unclear.

*Response new line 134: We visited each site in 2017 and 2019 once, never resampled.*

Line 129: Okay. So, this statement makes it sound like each site was only sampled once over the entire duration of the study. That means a site consists of sampling from a single time point and 5 or 10 4m2 plots. This totals, at most, 40m2 of sampling per data point. While there is no authority over what the minimum sampling effort for ticks should be, this seems very low and may explain why so few adults were collected over the course of the study.

*Response new line 136: Due to the longevity of I. ricinus ticks, a large amount of time is spent off the host, where the environment is posing a threat of survival of individual ticks (Mejlon, 2000). We believe that the tick life stage ratios in our study reflects the actual ratios in many areas and previous studies have found similar ratios with many larvae and nymphs and few adult ticks (Dobson et al., 2011; Ruiz-Fons et al., 2012). Due to the fact that we have sampled 5 – 10 plots per site the proportion of adult ticks is representative for the sample site, but very low in absolute numbers. Therefore, we agree with conclusion above from the reviewer.*

Line 135: It is unclear what local landcover the sampling occurred in. It is just referred to as greenspace here, but was it forest and if so what type? This is central to the second objective of the study.

*Response new line 142: Since we have randomly distributed our sampling sites in Stockholm County, our sampling sites consists of a quite large variety of different greenspaces. To analyze the effects of the local habitat, we have used the smallest buffer zone (100m) in the GIS analyses. In addition, to clarify we have restated the objectives of this study.*

Line 138: What is the snowball technique? Is that what is described in lines 138-144?

*Response new line 144: Instead of using the term snowball technique we are instead describing the methods how we are randomly selecting the different plots at each site.*

Line 154: How do you “cover” the activity patterns of ticks by only sampling each site a single time and having those single sampling occasions span from May to October?

*Response new line 161: See answer to comment #5*

Line 160: what is meant by the “height of the field layer”? what distance around the plot was used for stem density measurements? Was vegetation height/tree cover also measure in both 2017 and 2019, or only 2019?

*Response: The height of the field layer is the average vegetation height in each plot.*

*The tree cover density was measured using the Bitterlish technique which measures the number of tree stems adjusted for stem diameter at breast height and distance from the plot. In practice this means that we are counting small and large trees close to the plot but only large trees far away. The maximum distance for inclusion will differ depending in the size of the trees.*

*Vegetation height and tree cover were measured both in 2017 and in 2019 for each sampling plot.*

Line 179: include list of the eight forest subcategories here

*Response new line 187: We have added the eight forest subcategories used in this study to the methods section.*

Line 182: This size of the forest fragments that sampling sites were in would be an important variable to include in this analysis.

*Response new line 195: The effects of fragment size is included in the Contagion (CONTAG) variable.*

Line 184: Justification for why 1000m buffers were used for urbanization and landscape composition models is needed. One could perform an analysis to determine the appropriate ‘scale of effect’.

*Response: Landscape configuration is only important if individual habitat types is subdivided into several fragments or if the diversity of habitat types differs among sampling sites. These effects will most likely only be evident at the largest buffer zone size.*

Line 185: Was SHDI and contagion measured using the landcover classification with or without the forest subcategories?

*Response new line 197: SHDI and contagion were measured with all forest categories collapsed into a single category FOREST.*

Line 193: do you mean “To analyze the effect of possible risk factors….”?

*Response new line 203: Thank you pointing this out. We have revised the sentence.*

Line 210: It is unclear how spatial autocorrelation is accounted for within the models. Please explain and/or show results of tests for spatial autocorrelation.

*Response new line 222: In the statistical models, site specificity (using site as random factor) accounts for the correlation that may exist between ticks and the environment that may create a bias in the result from repetitive sampling at each sampling site. We have revised the sentence changing from spatial autocorrelation to spatial correlation to be clearer.*

Line 216: What aspect/measure of each land cover type is being used? Proportion?

*Response new line 229: Yes, we have used proportion of different land cover types in the different buffer zones.*

Line 216: How was this variation in sampling effort (5 vs 10 plots) accounted for in your response variable of abundance?

*Response new line 239: By the inclusion of sampling site as a random factor in the mixed models*

Line 219: Including the proportion of all landcover types in a single model is likely not appropriate as explanatory variables are very likely to be highly correlated given that the data is proportional. While the authors state that they tested for collinearity, these results are not reported, and the full models probably violated this rule. Please show VIF results and/or reduce variables included in full model.

*Response new line 233: The models are first reduced based on the significance levels and AIC values. The reduced model fit was assessed by the residual distribution of each model using package DHARMa and lastly tested for collinearity. We have added in the manuscript which variables that had to be removed from the models since they were highly correlated.*

Line 239: Need justification for why counts of nymphs and adults were combined. Because few adults were collected, it seems reasonable to just run a model for larvae and a model for nymphs.

*Response new line 255: The adults have been excluded from the statistical models.*

Line 277: Could the fewer ticks in taller vegetation be a detection issue?

*Response new line 294: We believe that the fewer ticks in taller vegetation is a negative effect of vegetation height and not detection issue. With the mopping technique, the mop handle permit easy adjustments to different vegetation heights. See discussion in the manuscript*

Table 1: Why so little consistency in significant landscape variables across scales for larval presence/absence and abundance?

*Response: We have reanalyzed the larvae models and kept the factor time to be present in all models (even though not significant). The same procedures were done for the models with nymphs. As a result, we can now see more consistency in significant variables across scales.*

Line 449: It is confusing to have a discussion of tick abundance under a section title tick presence and absence.

*Response new line 496: I think the title was tick presence and abundance.*

Figure 3: Add an inset map for spatial context and explain the difference between the white and black in the background.

*Response: Thank you for pointing this out. An inset map for the spatial context has been added as well as a legend for the black, white, and blue background.*

Thank you for all the time and comments from both the editor and reviewer.

Kindest regards,

Thérese Janzén

**References**

Dobson AD, Taylor JL, Randolph SE. Tick (Ixodes ricinus) abundance and seasonality at recreational sites in the UK: hazards in relation to fine-scale habitat types revealed by complementary sampling methods. Ticks and tick-borne diseases. 2011 Jun 1;2(2):67-74.

Mejlon H. *Host-seeking activity of Ixodes ricinus in relation to the epidemiology of Lyme borreliosis in Sweden* (Doctoral dissertation, Acta Universitatis Upsaliensis).

Ruiz-Fons F, Fernández-de-Mera IG, Acevedo P, Gortázar C, de la Fuente J. Factors driving the abundance of Ixodes ricinus ticks and the prevalence of zoonotic I. ricinus-borne pathogens in natural foci. Applied and environmental microbiology. 2012 Apr 15;78(8):2669-76.
