# Supplementary material for: Factors responsible for Ixodes ricinus presence and abundance across a natural-urban gradient
Source: PLoS One. 2023 May 17;18(5):e0285841. doi: 10.1371/journal.pone.0285841 (PMC10191369; doi:10.1371/journal.pone.0285841)
Supplement: Attachment — Submitted filename: Response to Reviewers.docx [file pone.0285841.s004.docx]

Janice L. Bossart
Academic Editor
PLOS ONE

PONE-D-22-15726R2

Factors responsible for *Ixodes ricinus* presence and abundance across a natural-urban gradient

PLOS ONE

Dear Dr. Bossart,

Thank you for letting us resubmit our manuscript to PLOS ONE. Your comments were highly insightful and have improved our manuscript. Attached are our responses to your comments.

We have added a paragraph to the discussion that addresses how the lack of data for adult ticks’ factors into our understanding of *Ixodes ricinus* presence and abundance across the urbanization gradient. In our study, adult ticks were absent in highly urbanized areas. Urbanized greenspaces that are surrounded by build environments generally have a reduced diversity of host animals, especially larger mammals on which adults’ feed. The few adult ticks collected in this study were sampled in suburban forests and at these sites other tick life-stages were also found. These suburban forests have favorable habitats for ticks and hosts and here all tick life stages can find a host for their blood-meals.

We have also reworked the results section which is now presented with interesting patterns explaining *I. ricinus* presence and abundance. Lastly, we have worked through the manuscript, which is now tightened up and clear and to the point. All changes to the manuscript have been marked with yellow color.

Below is our point-by-point response to each of your comments.

On behalf of all the authors

Sincerely,

Thérese Janzén

Corresponding author

Point-by-point responses

As far as I can tell, 10 plots/site were sampled in 2017 and 5 plots/site were sampled in 2019.  If I have that right, then for clarity simply just state that rather than using verbiage like '*Line 245-249:* were sampled'.

*Response: Accepted*

What factors are local site/scale factors?  In the M&Ms (236-237) you list vegetation height, tree stem density, and temperature.  But then in Table 4, you indicate coniferous forests. Could Tables 1&2 be divided into local scale factors vs all others? Do these Tables only include factors that are significant? I'm assuming so, so please explicitly state that in the caption.

*Response: We have divided up table 1 and 2 in local site factors (100m) and surrounding landscape (200-1000m). Table 4 we have divided into local plot, local site and surrounding landscape.*

Numbering and discussion order of Tables & Figures should correspond. Currently, you cite out to Table 4 before Table 3. Either change the order of their discussion or the numbers of the tables.

*Response: Accepted*

In the text *Time* is identified as a factor but the Tables don't list *Time* as a factor, they list *Month*.  Choose either Time or Month.

*Response: Accepted*

Regularity of sampling is still unclear.  Did you sample weekly? bi-weekly? daily? 3 days in a row? some other way?  Please clarify.

*Response: Our sampling has been irregular and therefore we have added the time factor Month into our models. We have added information regarding the regularity of our sampling to our manuscript.*

*Line 135: Field sampling for both years occurred irregularly with most sampling times in June and August.*

*Line 245-249: The second order polynomial of Month indicating inventory time (sampling Day caused convergence issues) was added to correct for the irregular sampling times. In addition, sampling site was used as random variable to adjust for the difference in sampling effort between sites, and to handle the correlative structure among plots within the same sampling site in the conditional part of the model.*

Line 70: N. Europe is a misnomer given you only looked at one spot in N. Europe, i.e. Stockholm County

*Response: Accepted*

Line 84: Delete the period after 'sites'

*Response: Accepted*

Line 105: Change 'picture' to 'figure'

*Response: Accepted*

There are many spots where verb agreement is incorrect, e.g. Line 105: 'show' should be 'shows', line 135 'were' should be 'was', line 137 'allow' should be 'allows', line 222 'account' should be 'accounts', line 291 'was' should be 'were', line 316 'represents' should be 'represent', etc. Given how many I uncovered just quickly reading through certain sections, I'm guessing there are others. Please carefully scrutinize all text to ensure verb usage is correct.

*Response: We have carefully scrutinized all text.*

Line 130: Insert 'them' after 'excluded'

*Response: Accepted*

Line 222: delete either 'these' or 'the'

*Response: Accepted*

Lines 232-238: This is a really long sentence.  Rather than having 'fixed factors' at the end, how about flipping the text around to something like, Fixed factors included...  Then list the factors.

*Response: Accepted*

Line 239: Add 'a' before 'random'

*Response: Accepted*

Line 256: Replace 'but not for adult ticks' with 'and excluded adult ticks from the analyses'.

*Response: Accepted*

Line 273: Replace existing with, "Adult ticks were excluded from all statistical analyses". (I was back and forth on whether this should only be here or only in M&Ms or in both, and decided it seemed best in both locations)

*Response: Accepted*

Lines 282-284: Delete and add as footnote to the Tables.

*Response: Accepted*

Line 285: 'provide' should be 'provided'

*Response: Accepted*

Lines 290-292: If these were the only local factors (as indicated in lines 236-237), then how about just simply state something like, All local site factors, i.e. veg height, tree stem density, and temp, all had a significant effect on...

*Response: Accepted*

Examples of M&Ms included in the Results: Lines 273-274, 280-281, 292-295, 314-316, etc. Please carefully scrutinize the Results section and ensure that M&Ms are in the M&Ms section where they belong.

*Response: Accepted*

Line 275-277: Since 273-274 will be removed since it's not results, simply reword to something like, Highly correlated variables removed from the analyses of landscape configuration...include...  etc.

*Response: Accepted*

Line 318: 'lager' should be 'larger'.

*Response: Accepted*
