# Supplementary material for: Factors responsible for Ixodes ricinus presence and abundance across a natural-urban gradient
Source: PLoS One. 2023 May 17;18(5):e0285841. doi: 10.1371/journal.pone.0285841 (PMC10191369; doi:10.1371/journal.pone.0285841)
Supplement: Attachment — Submitted filename: Response to Reviewers.docx [file pone.0285841.s005.docx]

Janice L. Bossart
Academic Editor
PLOS ONE

PONE-D-22-15726R2

Factors responsible for *Ixodes ricinus* presence and abundance across a natural-urban gradient

PLOS ONE

Dear Dr. Bossart,

Thank you for letting us resubmit our manuscript to PLOS ONE. We took your advice and asked someone who was unfamiliar with our manuscript and who works as an academic writer read it through carefully. All changes to the manuscript have been marked with yellow color.

Below is our point-by-point response to each of your comments.

On behalf of all the authors

Sincerely,

Thérese Janzén

Corresponding author

Point-by-point responses

Line 84-85.  Delete the 2nd 'in 2019'

*Response: Accepted*

Line 199. Add a comma after 'attributes'

*Response: Accepted*

Line 278. Remove 'that'

*Response: Accepted*

Line 284. 'Curve linear' should be 'curvilinear'

*Response: Accepted*

Line 307. Capitalize 'spruce' for consistency

*Response: Accepted*

Line 403. 'dryer' should be 'drier'

*Response: Accepted*

Line 406. 'steam' should be 'stem'

*Response: Accepted*

Line 465. Add a comma after 'stage'

*Response: Accepted*

Line 467. 'build' should be 'built'

*Response: Accepted*

Table 2 Heading. 'Ricinus' should be lower case

*Response: Accepted*

Table 4 Heading. Should be 'Landscape features include...'

*Response: Accepted*

Please italicize all scientific names.  The vast majority of these are *Ixodes ricinus*, but there are a few others.

*Response: Accepted*
